# Supplementary material for: Effect of Fluridone on Roots and Leaf Buds Development in Stem Cuttings of Salix babylonica (L.) ‘Tortuosa’ and Related Metabolic and Physiological Traits
Source: Molecules. 2024 Nov 16;29(22):5410. doi: 10.3390/molecules29225410 (PMC11597348; doi:10.3390/molecules29225410)
Supplement: Supplementary file 1 [file molecules-29-05410-s001.zip › molecules-3283922-supplementary.pdf]

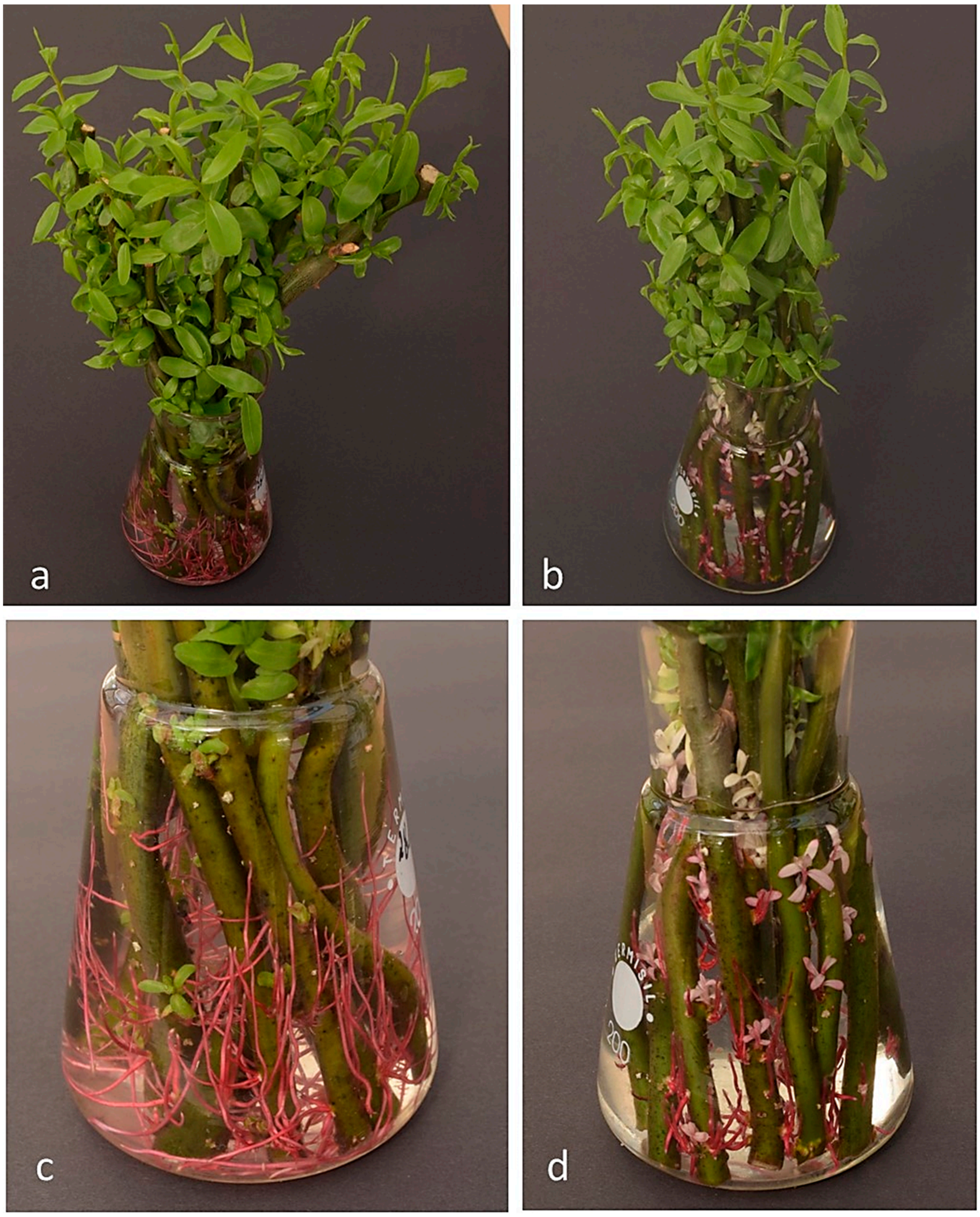

Figure S1. Effect of fluridone on adventitious root development in *Salix babylonica* stem cuttings after whose lower parts were placed in water water (a,c) and fluridone solution (10 mg/L) (b, d). The pictures were taken after 2 weeks of experiment

Table S1. Content (μg/g DW ± sd) of anthocyanins in leaf buds of *S. babylonica* collected from cuttings over a layer of water or fluridone solution. Means in the rows marked with the same letter do not differ at the significance level of p = 0.05 according to Duncan's test

**Anthocyanin**	**Control, buds above water layer**	**Fluridone, buds above solution layer**
Cyanidin glucoside	0.55 ± 0.05[a]	0.30 ± 0.05[a]
Cyanidin galactoside	7.6 ± 0.3[a]	6.6 ± 0.2[a]
Cyanidin rutinoside	1.4 ± 0.2[a]	1.2 ± 0.1[a]
Delphinidin glucoside	246.9 ± 5.0[a]	233.5 ± 3.0[a]
Delphinidin rutinoside	8.8 ± 0.2[a]	7.4 ± 0.3[a]
Delphinidin acetyl-glucoside	89.6 ± 1.2[a]	82.4 ± 2.5[a]
Pelargonidin glucoside	4.6 ± 0.4[a]	3.4 ± 0.3[a]
Petunidin glucoside	0.36 ± 0.02[a]	0.43 ± 0.02[a]
Malvidin glucoside	0.51 ± 0.02[a]	0.46 ± 0.02[a]
Peonidin glucoside	23.3 ± 0.4[a]	22.9 ± 0.3[a]
Peonidin rhamnoside-glucoside	0.34 ± 0.05[a]	0.36 ± 0.02[a]
Total antocyanins	384 ± 7.8[a]	359 ± 5.5[a]

Table S2. Content (μg/g DW ± sd) of salicinoids in leaf buds of *S. babylonica* collected from cuttings over a layer of water or fluridone solution. Means in the rows marked with the same letter do not differ at the significance level of p = 0.05 according to Duncan's test

**Salicinoid**	**Control, buds above water layer**	**Fluridone, buds above solution layer**
Helicin	0.27 ± 0.01[a]	0.31 ± 0.01[a]
Tremuloidin	4.73 ± 0.10[b]	5.33 ± 0.07[a]
Salicin	64.5 ± 0.9[a]	59.4 ± 0.8[b]
Salicortin	193.8 ± 5.1[b]	229.2 ± 2.6[a]
Total salicinoids	263 ± 6.1[b]	294 ± 3.4[a]

Table S3. Content (μg/g DW ± sd) of total contents, free forms (F), esters (E), glycosides (G) of flavonoids in leaf buds of *S. babylonica* collected from cuttings over a layer of water or fluridone solution. Explanation of abbreviations: F – free form; E – esterified form; G - glycosidic form; tr –traces. Means in the rows marked with the same letter do not differ at the significance level of p = 0.05 according to Duncan's test

**Flavonoid**		**Control, buds above water layer**	**Fluridone, buds above solution layer**
Prunin		0.22 ± 0.01[a]	0.23 ± 0.03[a]
Taxifolin		0.04 ± 0.01[a]	0.04 ± 0.01[a]
Quercetin			
	total	0.52 ± 0.06[b]	1.38 ± 0.05[a]
	F/E/G	0.21/0.27/0.04	0.46/0.87/0.05
Apigenin			
	total	0.81 ± 0.08[a]	0.94 ± 0.02[a]
	F/E/G	0.23/0.04/0.53	0.18/0.05/0.71
Kaempferol			
	total	0.61 ± 0.02[a]	0.63 ± 0.06[a]
	F/E/G	0.12/0.24/0.25	0.09/0.25/0.29
Epicatechin			
	total	0.08 ± 0.02[a]	0.07 ± 0.01a
	F/E/G	0.08/tr/tr	0.07/tr/tr
Total flavonoids		2.28 ± 0.20[b]	3.29 ± 0.18[a]

Table S4. Contents (μg/g DW ± sd) of total contents, free forms (F), esters (E), glycosides (G) of phenolic acids in leaf buds of *S. babylonica* collected from cuttings over a layer of water or fluridone solution. Explanation of abbreviations: F – free form; E – esterified form; G - glycosidic form; tr – traces. Means in the rows marked with the same letter do not differ at the significance level of p = 0.05 according to Duncan's test

**Phenolic acid**		**Control, buds above water layer**	**Fluridone, buds above solution layer**
Ferulic			
	total	0.64 ± 0.04^{a}	0.66 ± 0.03^{a}
	F/E/G	0.02/0.62/tr	0.01/0.65/tr
p-Coumaric,			
	total	11.32 ± 0.58^{a}	11.16 ± 0.31^{a}
	F/E/G	0.33/10.96/0.02	0.19/10.95/0.02
3-hydroxy-Benzoic,			
	total	0.28 ± 0.02^{a}	0.24 ± 0.02^{a}
	F/E/G	0.02/0.14/0.12	0.02/0.14/0.08
Protocatechuic,			
	total	1.09 ± 0.07^{a}	1.12 ± 0.08^{a}
	F/E/G	0.15/0.12/0.82	0.11/0.17/0.84
Caffeic,			
	total	12.95 ± 0.09^{a}	10.82 ± 0.30^{b}
	F/E/G	0.55/12.40/tr	0.29/10.53/tr
Total phenolic acids		26.28 ± 0.80^{a}	24.00 ± 0.74^{a}
